# Older Black Lesbians’ Needs and Expectations in Relation to Long-Term Care Facility Use

**DOI:** 10.3390/ijerph192215336

**Published:** 2022-11-20

**Authors:** Mekiayla Singleton, Mary Anne Adams, Tonia Poteat

**Affiliations:** 1 Leonard Davis School of Gerontology, University of Southern California, Los Angeles, CA 90089, USA; 2 ZAMI NOBLA: National Organization of Black Lesbians on Aging, Atlanta, GA 30364, USA; 3 Department of Social Medicine, University of North Carolina at Chapel Hill, Chapel Hill, NC 27599, USA

**Keywords:** sexual minorities, care plans, LGBT, person-centered care

## Abstract

There is a dearth of long-term care research that focuses on the expectations and experiences of older sexual minority (SM) adults. That research dwindles further when examining subgroups within that population such as older Black lesbians. The purpose of this study was to explore older Black lesbians’ needs and expectations in relation to the utilization of long-term care (LTC) facilities. We conducted secondary data analysis using data from 14 focus groups that discussed health and aging with older Black lesbians. Transcriptions were analyzed in NVivo using deductive content analysis and structural coding. Three themes were identified in relation to needs and expectations for LTC facility use: (1) consideration or established plans to utilize a LTC facility, (2) concern for care facility environment, and (3) a desire to build one’s own community. These findings illustrate how older Black lesbians are planning for a potential need for LTC, their concerns about utilizing LTC, and alternative approaches to avoid LTC use. There remains a continued need for LTC communities that are inclusive and supportive of SM older adults as well as more SM-only communities where older adults can live openly and authentically.

## 1. Introduction

Approximately two in five sexual and gender minority (SGM) older adults are people of color with Native (28%), Black (19%), and Latino/a (14%) identities making up the largest portions [1,2]. Given this diverse makeup and that the older adult SGM population is continuously growing [3], there needs to be continued research to better understand the aging experiences of racial and ethnic older SGM adults. Chen and colleagues [4] noted that the aging experiences of racial and ethnic SGM older adults included experiences of stigma and discrimination, isolation, lack of support, and uncertain futures. While these issues are common among the general older adult SGM population, there are social structures and systems that exacerbate these issues for those in a racial and ethnic minority group.

Although racial and ethnic minority SGM older adults have heightened concerns around care and support as they age, there are disparities around these concerns between groups. A 2018 AARP report found that older Black SGM individuals were more concerned about multiple forms of discrimination and negative outcomes than White or Latino SGM individuals [5]. Forty-two percent of older Black SGM respondents reported being “somewhat or very concerned” that the quality of care received would be impacted by their race in addition to 38% that had the same concern for their sexual orientation. Being “very or extremely concerned” about having adequate family and/or social support as they age was reported at higher rates among older Black SGM respondents at 56% compared to 49% and 39% for Latino and White SGM respondents, respectively [5]. These outcomes highlight how older Black SGM adults have increased aging-related concerns compared to their peers.

Given that older Black SGM adults have expressed heightened aging-related concerns, it is important to investigate what those specific concerns are and their expectations about aging. Since the Black SGM experience is not monolithic, it is critical that the subgroups within this population are centered to better address their needs. One such group is Black lesbians. Older Black lesbians face several health disparities that include an increased risk of disability, obesity, and mental health issues [6,7]. These disparities are related, but not limited to their higher likelihood to have experienced discrimination and stigma, socioeconomic disadvantage, and a lack of access to adequate healthcare [8,9,10,11]. Although older Black lesbians have these increased health risks as they age, there remains a gap in understanding what other aging-related issues they may have. This is particularly the case regarding experiences of long-term care (LTC) and other support services.

A common concern for Black lesbians related to growing older includes potential loss of physical and financial independence and becoming a burden to others [10,12]. In a qualitative study which discussed long-term care options with Black lesbians, having long-term care insurance, and being in a facility with a community of other Black lesbians was of the greatest importance [12]. However, a gap remains in understanding other needs and expectations of older Black lesbians in relation to long-term care, such as facility use. Previous research has yet to explore why older Black lesbians may need LTC, what plans they may already have in place, and the potential concerns in utilizing LTC. Using qualitative data, this study aims to explore what those needs, and expectations may be.

## 2. Materials and Methods

### 2.1. Researcher Reflexivity

It is important that scholars engaged in critical social science research consider how their position influences their interpretation of people’s lived experiences [13]. Keeping this in mind, the first author (Singleton) acknowledges her position as a young, straight Black woman and how those identities, particularly her age and sexual orientation, may influence her analysis of the data. Prior to Singleton taking the lead in the secondary qualitative analysis, she and the second author (Adams) had an extensive discussion on her interest in aging issues as a young person and her focus on these issues within the SGM population. In this conversation, Singleton disclosed her educational and professional work with the older adult population and those within the SGM community.

Mary Anne Adams has expertise in social work, community-based research, public health and health disparities. Dr. Tonia Poteat’s expertise and research focuses on HIV and SGM health disparities. Both co-authors (Adams and Poteat) identify as Black lesbians and were responsible for the conceptualization and data collection of the original study that this data comes from. Given this, it was important that all the researchers established a foundation of trustworthiness and accountability. Throughout the data analysis, Ms. Adams and Dr. Poteat provided extensive feedback, including addressing any observations of ageism and homophobia that may have presented itself. It is through this process that the data was able to be handled with the utmost care and attention.

### 2.2. Data

The data for this study were drawn from transcripts created during a prior qualitative study of older Black lesbians and aging. The intention of the primary study was to gather information about the health and aging needs of older (40+ years of age) Black lesbians [14]. This data was collected in 2014 in metropolitan Atlanta, Georgia, where participants were recruited through networks of key gatekeepers and Black SGM organizations. Inclusion criteria for the original study were: (1) being a woman aged 40 and older, (2) residing in the metropolitan Atlanta area, (3) identify as Black and/or African descent, and (4) identify as lesbian, same-gender loving, or gay. Fourteen focus groups were conducted with a total of 100 Black lesbians who were aged 41 to 91 years old. Each focus group lasted around 1 ½ to 2 hours and participants were asked a series of open-ended questions about aging and healthcare needs. Some examples of the open-ended questions asked were “what have you noticed as you’ve gotten older?”, “has anything surprised you about getting older?”, “how would you describe a healthy, Black lesbian?”, and “if the Black lesbian community had unlimited funding to do anything, what intervention would you like to see?” [14]. Prior to the start of each focus group, verbal informed consent was obtained for each participant. Institutional Review Board approval was provided by the institution at which the original study took place. Table 1 shows the participant demographics.

### 2.3. Study Design and Analysis

**Figure 1:**
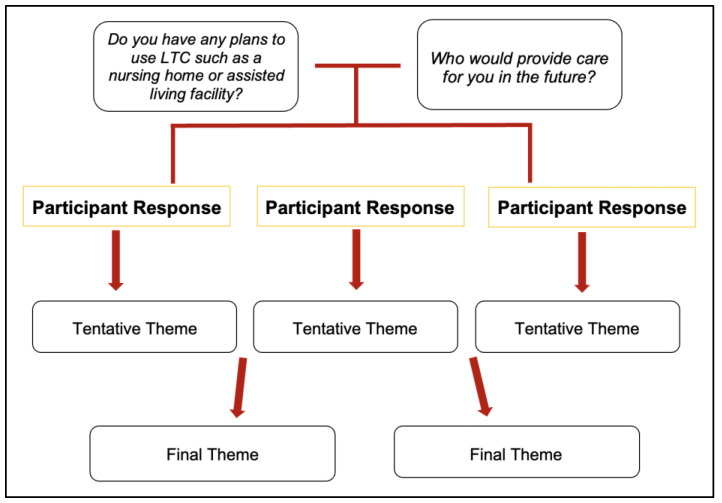
Coding Illustration.

The transcriptions from the focus groups were uploaded to NVivo 12 for secondary analysis. The first author (Singleton) conducted an initial content analysis [15] on the focus group transcripts to ascertain whether the topic of LTC and other related issues were mentioned or discussed. This first round of analysis was important because the intent of the original data collection was to gather information on possible interventions to improve the health and aging needs of older Black lesbians. [14]. The results of the initial content analysis found that participants were asked questions such as “*Who would provide care for you in the future?”* and “*Do you have any plans to use LTC such as a nursing home or assisted living facility?”*. Noting these questions and their alignment with the research question, the first author then went through each transcript and coded the data utilizing a *structural coding* method. Structural coding is a form of qualitative coding that involves coding the data for information that can answer the research question or topic of interest [16]. For this study, most responses to the above-mentioned questions were coded and grouped into tentative themes. Themes were created based on a group of responses that all had a common idea, thought, or sentiment expressed. These tentative themes along with the coded responses were shared with the co-authors, who aided in the revision process until 100% consensus was reached. An example coding illustration is shown in Figure 1.

## 3. Results

Three themes were identified in relation to needs and expectations for LTC facility use: (1) consideration of or established plans to utilize a LTC facility, (2) concern for care facility environment, and (3) a desire to build one’s own community. Within these themes, participants discussed issues such as having to rely on LTC due to lack of family or social support, the possibility of being isolated and hiding their lesbian identity and creating communities of mutual support to avoid facility-based care. In the following sections, we define each theme and provide exemplar quotations from participants.

### 3.1. Consideration or Plans to Utilize Long-Term Care (LTC)

This category involved participants discussing the possibility that they might need LTC and already having plans in place if that were to happen. Several of the women mentioned that they would consider moving to a nursing home, an assisted living facility, or both. Some women stated that moving to a care facility would be the next step when asked who would care for them if they were no longer able to care for themselves:

*“Basically, most of my family is gone, so I would stay in a nursing home”*

*(**Person 1, Group 10**).*

*“I don’t know. I think I would have to do an assisted living type of facility”*

*(**Person 4, Group 13**).*

*“I guess I’ll be in somebody’s home some kinda way somewhere. I don’t know”*

*(**Person, 6, Group 6**).*

Similar to those who are non-sexual minorities, for those who did have children or family, they mentioned not expecting them to be the ones to take care of them or already knowing that their family will try to put them in a facility. One participant said:

*“Well, I have children and grandchildren. But my grandchildren, they’ve already informed me that although they love me, they will try to make enough money to put me somewhere for somebody to see after me. They’ve already told me”*

*(**Person 7, Group 10**).*

Another expressed similar sentiments; however, she further stated not really having an idea of what is next:

*“I have to agree with [participant]. I have no children [or a] partner. And that does weigh on me at my age right now, as to what would be my next step…I have brothers. I have nieces, but it’s not anything that I’m gonna try to expect from them or the children. It’s just something that I’m trying to mentally grasp, what is that next step. It’s troubling because you just don’t know”*

*(**Person 2, Group 13**).*

There was one participant who stated she would move to a nursing home because she did not trust her family:

*“I wouldn’t trust anyone in my family to take care of me. So, it would definitely be a nursing home. That about sums it up”*

*(**Person 9, Group 10**).*

She goes on further to state that she does not have a problem with being in a nursing home as long as she is able to “get out from time to time” and not have “to be around those people all day long”. In this example, she expresses her contentment with being at a facility but still wanting to have some level of independence.

Furthermore, some women discussed financial plans they have established in order to afford LTC when the time comes. Long-term care insurance was specifically mentioned by a few participants as a plan to pay for the additional care they may need:

*“I thought about growing old and not being able to care sufficiently for myself. I have both a long-term care insurance plan that I think is comprehensive and should serve me well when that time comes if I can’t continue to afford to pay for it”*

*(**Person 1, Group 14**).*

*“I’ve tried to plan financially like to have good insurance to go to a nursing home or something like that”*

*(**Person 2, Group 5**).*

*“I’ve got to get some long-term care insurance and put some things in place for me”.*

*—**Person 7, Group 5***

In myriad ways, older Black lesbians have considered or have made plans to utilize LTC in the future. While many of the women have expressed their reasons for why they are considering LTC, some have also expressed concerns about their LTC options.

### 3.2. Concern for the Facility Environment

The environment in which care is provided and received is a major concern for those in the SGM community. Many older SGM adults have valid concerns that revolve around threats of discrimination and mistreatment when considering moving to long-term care facility [17,18]. These same sentiments were expressed by several participants when asked if they have considered moving to a LTC facility. The women noted wanting to be at a place that was “lesbian sensitive”, being fearful of losing one’s sexual identity and who they authentically are and losing independence. One participant shared:

*“So, I think that that would kill me, sort of softly kill me to have to live in a situation where all of a sudden, couldn’t be authentically who I was. I think it’s more real than we realize ‘cause not that many people are disclosing because there’s a trust issue and they don’t know who they’re telling and who you’re gonna tell. I think we probably encounter people all the time that are choosing to live in the closet for survival and we just don’t even know it”*

*(**Person 8, Group 5**).*

The message expressed here highlights the potential damage that non-disclosure can have on someone who may need to receive care from a place that is not a welcoming environment. Another participant made a similar comment:

*“But even if I did sell my home and move into a facility, would they be lesbian sensitive? That really bothers me. I don’t want to have to at this age, kinda of suppress that. So that makes me gravitate toward staying in my home and do what I wanna do”*

*(**Person 11, Group 1**).*

One participant stated the cumulative effects of moving to a facility as a lesbian:

*“From what I’ve seen of nursing homes and assisted living, it’s just horrible. It’s this loss of independence first and foremost and just loss of everything that you have worked your whole life to build… and I’m not just talking about your possessions, but who you are. So, I just cannot imagine. I’ve read a lot about LGB folks going into facilities and basically losing who they are, losing their life and their identity and that’s a scary thing. It’s a scary thing first of all going into assisted living or a nursing home for me, just the thought of it, and then on top of that, to lose myself”*

*(**Person 10, Group 5**).*

These comments made by multiple women in the focus groups acknowledge the major concern that many SGM adults have regarding moving to a facility. There is an overarching sense that these facilities are not SGM friendly and that in order to live in these types of communities you would have to stifle and hide your true self.

An interesting perspective that was brought up by a couple of the women were the systemic issues within facilities such as nursing homes. A **participant from group 14** said this when asked if she would consider a nursing home if the staff and resident identified similar to her, *“I don’t know because it doesn’t matter if you’re lesbian or straight, it’s your character. It’s like how do you know that just because they’re in the same demographics that you are that they’re gonna take care of you like your family would or your child would”.* Another person in the same group took this same issue a step further by commenting on the lack of resources provided to nursing homes. She reflected on her time as a nurse working in a nursing home and how overwhelming it was because she was given a large caseload:

*“I just want to address the whole thing about nursing homes. I don’t know if it’s so much the character of the people who work there, although of course that’s a part of it, it’s the resources that we give to facilities like nursing homes. They’re just not adequate. I remember working at one as a nurse. I don’t know if I was a nurse or an aid, but I remember they gave me like twenty-something patients and I’m like what the hell am I supposed to do. So even if you have an LGBT nursing home, without adequate resources you’re still not gonna have adequate care. So that I think has gotta be addressed on a systemic level, is how are we treating our elders and if you’re lucky, we’ll all be old one day*

*(**Person 11, Group 14**).*

The lack of adequate resources is an issue that many LTC facilities face [19]. As mentioned by some of the participants, without adequate resources no facility—SGM friendly or not—will be able to provide adequate care to older adults. This overarching problem of facilities not being able to provide proper care is another issue that individuals must consider when choosing to use LTC.

### 3.3. Building A Community of Mutual Support

As many of the women expressed their concerns with LTC facilities, it led to discussions of what they desire as they age: creating their own community. Due to the lack of SGM-specific facilities, specifically lesbian homes or communities, the women discussed the desire to form these types of communities that provide mutual support, help combat isolation, and even encourage intergenerational connection. One participant stated:

*“I’ve been researching lesbian homes or lesbian community… and there aren’t any in the U.S. I’ve been trying to get with some of my real estate investor friends and things like that, my older friends and say, “Look, what are you doing? What are we doing? You have property over here…. let’s combine those efforts and see if we can create a community. So that’s my effort for myself, is to whatever it is I like, I wanna create it. So, I want to get those people involved so I’m not taking care of her, she’s not taking care of me, but we’re supporting each other on some sort of mutually invested interests. And then I’m assured to be around those people that I’ve cared [for] and cultivated a relationship with. So that’s what I’m doing right now. I’m building that sort of community”*

*(**Person 4, Group 6**).*

Building a supportive community is critical for these older Black lesbians. Many understand that not everyone has biological family they can rely on and that it is important to create these deep and vested relationships in hopes that the community created will continue to exist. Two other participants further expressed the importance of having a community:

*“I think intentional communities. I have a lot of friends. We’re talking about starting an intentional community which I also think will help with growing older together and supporting one another that way as well. I think that’s really important. Everyone’s not going to have partners to take care of them. Everyone’s not going to have children to take care of them”*

*(**Person 8, Group 1**).*

*“I think the loneliness and isolation piece is very relevant to this topic because as lesbians age, many lesbians don’t have children. Many have been ex-communicated from their family of origin…so the fact of the matter is, the thought of aging and being without family or without community, it’s a very real topic. We talk about building community and I think that’s really critical. I have one of my very good friends, she doesn’t have any family [or] children. I’m concerned about her aging and being alone. I would take her into her house and take care of her if I needed to, but I have a partner, so how does that really work out?”*

*(**Person 3, Group 8**).*

This importance of having a community also was discussed when the women were asked who would provide care for them if they were no longer able to care for themselves.

*“I’m single without a partner. So, I have two best friends. We had this conversation a couple of times about us living in community and trying to take care of one another”*

*(**Person 6, Group 10**).*

*“Hopefully my community. I’m a part of a Yoruba community, Santeria community, and family member, nieces and nephews. I don’t have a partner and I’m not looking for a partner so it would be my community”*

*(**Person 3, Group 12**).*

*“My partner, children, lifelines. I’m building community now. You have to build community. You wanna believe that everyone’s gonna be on board or even around. You make assumptions that people will be there to take care of you and what if they’re not there, or what if they’re unable to. So, you might need to build community to corral not just for yourself but for each other”*

*(**Person 5, Group 12**).*

*“I have strong family relationships with my sisters’ children, my nieces. I think they would care for me, but in addition to all that, my partner and I have started dialogue with friends who are of various ages where we are thinking more about caring for each other and being each other’s support. I feel pretty confident about getting older”*

*(**Person 6, Group 14**).*

For these women, the message is clear. Surrounding yourself with people that you trust and can be your authentic self with is most important in considering what your future care needs may be. It is much preferred to be cared for by a community that you have built and where there is mutual trust.

## 4. Discussion

This present study highlights what older Black lesbians’ needs and expectations are around LTC facility use. Many of the older Black lesbians in this study acknowledged their needs and plans for moving to a LTC facility, their expectation of the facility environment, and their desire to create alternative communities of mutual support. The findings from this study contribute to the scant literature about the aging experiences of Black lesbians [9,11,12,14,20] and demonstrate parallel sentiments around aging and LTC use for the larger, SGM older adult population [17,21,22,23].

Many of the older Black lesbians in this study mentioned plans to utilize LTC in the future. When asked who would provide care for them if they could no longer care for themselves, some women anticipated the need for LTC due to their lack of family support and not having any LTC plans. A lack of family and social support is a major aging concern for those in the SGM community, particularly those who are a racial and/or ethnic minority [5]. Additionally, prior literature has shown that those without children report a higher anticipated need to use a LTC facility, such as a nursing home, in the future [24]. However, those who did have family support acknowledged not expecting them to provide care thus leading them to anticipate and consider utilizing a LTC facility. An additional layer to the discussion around LTC use was financial planning. A few of the women had obtained LTC care insurance in anticipation of perhaps one day needing such accommodations. Prior research on Black lesbians showed that LTC insurance was a major aging concern, particularly when it came to where they could use said insurance [12]. Transitioning to Black lesbian-specific communities is a top priority; however, this may not be possible given the dearth of such communities, thus leading to a greater concern around the likelihood of non-friendly lesbian environments at LTC facilities [12].

A key issue mentioned in this study was a concern for the LTC facility environment. The prior literature has noted the expectation that older SGMs have of anticipatory discrimination, mistreatment, and discomfort in LTC settings [17,21,23]. When planning or considering the need for LTC, many older SGMs expect these environments to be harmful and not welcoming and inclusive of diverse individuals [5]. An important aspect in this study was that the women expressed the fear of losing the connection to their lesbian identity. This fear stemmed from having read and heard about how sexual minority people are treated in LTC and not wanting to be in a discriminatory and unsafe environment that would force them to hide their true selves. Additionally, prior research has shown that older SGM adults of racial and ethnic minorities share a similar fear of discrimination and unfair treatment in LTC facilities based on their racial/ethnic background and in conjunction with their sexual identity [5]. Being able to openly and freely claim one’s sexual identity is an issue that many in the SGM community are concerned with as they age [25].

In addition to the concern for facility environment regarding one’s sexual identity, a point was made about the adequacy of the care received in LTC facilities. Some participants addressed the issues of LTC facilities on a systemic level; the lack of necessary resources to provide quality care. The quality of care received in LTC facilities, such as nursing homes, has an extensive history of being substandard and can be largely attributed to the understaffing of nurses [19]. Consequently, some residents in these facilities are subject to more physical injuries, emergency room visits or hospitalizations, and other medical issues [19]. This is a major concern for anyone who considers LTC use, and these issues continue to be prevalent given the current COVID-19 pandemic [26,27]. For these older Black lesbians, they understand and share common sentiments with those outside of their community about the systemic issues that may impact the quality of care received in a LTC facility.

Some SGM older adults seek community support in place of familial assistance. For some, their geographical location may place them far from necessary resources [28]. The older Black lesbians in this study expressed the desire to create communities of mutual support to have in place before crises occur. Although community support is of importance to SGM older adults in general, it is critical to note that the communities the women in this study are speaking about also consist of other Black lesbians. They understand the many disadvantages that some Black lesbians face as they get older (i.e., no children, estranged from family, lack of partner) as well as the lifetime experience of discrimination and stigma (as a result of racial and sexual identity) and want to impede the issues of isolation and loneliness [20,29,30,31].

The outcomes from this study have policy and practice implications. Noting the multitude of reasons that older Black lesbians in this study anticipate needing LTC, facilities should be equipped with the resources and knowledge necessary to provide quality, person-centered care. A starting point for this could be facilities undergoing regular, long-term cultural humility training designed to promote equitable and inclusive care to SGM older adults in residential LTC that is facilitated by LGBTQAI+ organizations. Another critical implication is the greater need for LTC communities and facilities that are specifically built for the SGM community. As mentioned above, many of the older Black lesbians in this study envision their future care consisting of mutual support whether it be through their own network or moving to an already established community. It is imperative that future funding and resources are allocated to create such spaces for individuals of diverse and marginalized backgrounds to age safely and authentically.

### Limitations

One limitation of this study is the time and place in which this data was collected. This data was collected in 2014 in the Atlanta, Georgia area. The outcomes from this study are specific to the participants who took part in the focus groups, precluding our ability to generalize these findings to the entire older Black lesbian population. Another limitation of this study is that the aims differed from the original study. Thus, the discussions around LTC facility use that occurred in these focus groups may not have included all the needs and expectations that older Black lesbians may have.

## 5. Conclusions

The outcomes from this study provided some key takeaways when it comes to LTC facility use and older Black lesbians. Many of the women in this study anticipate a need to use LTC for a multitude of reasons. Given this, it is imperative that facilities that provide such care are equipped with the resources necessary to provide quality, person-centered care to a diverse population. Although some women in this study expressed future LTC use, others acknowledged their concerns with being in those environments. These concerns lead to what many of the women in this study desire: to build a community of mutual support. For this group of older Black lesbians, the goal is to be cared for by one another and to be in a community that allows them to be authentically themselves. Future studies should aim to specifically address other long-term care needs of SGM subgroups and ways that these communities might address those issues.

## Figures and Tables

**Table 1:** Demographics of Participants.

	N = 100
Age *	54
**Ethnicity**	
African American	95.0
Caribbean/West Indian	6.0
Hispanic	2.0
Bi-Racial/Multi-racial	7.0
Other	2.0
**Sexual Identity**	
Lesbian	81.0
Bisexual	3.0
Gay	8.0
Same Gender Loving	14.0
Other	2.0
**Education Level**	
Some High School	2.0
High School Graduate/GED	14.0
Some College	30.0
College Graduate	29.0
Graduate/Professional Degree	24.0
**Religion**	
Protestant	61.0
Catholic	2.0
Muslim	1.0
Buddhist	4.0
Other	23.0
**Relationship Status**	
Single	43.0
Partnered	51.0
Other	6.0
**Marital Status**	
Legally married to man	3.0
Legally married to woman	10.0
Civil union with man	0.0
Civil union with woman	8.0
Registered domestic partnership	6.0
Common-law marriage	0.0
Legal separation	2.0
Divorced	18.0
Never married	38.0
Other	14.0
**Household Members**	
Self	31.0
Self and spouse	3.0
Self and partner	27.0
Self, spouse/partner, and family members	14.0
Self and family members	18.0
Roommate	6.0
**Occupation**	
Unemployed	29.0
Employed	58.0
Self-employed	4.0
Retired	7.0
**Health Insurance**	
Uninsured	20.0
Insured w/private	37.0
Insured w/government	17.0
Insured w/Medicare and/or Medicaid	27.0
**Household Income**	
<$10,000	13.0
$10,001 to $30,000	23.0
$30,001 to $60,000	39.0
$60,001 to $100,000	16.0
>$100,000	5.0
**Health Rating**	
Poor	1.0
Fair	20.0
Good	50.0
Very Good	1.0
Excellent	2.0
Other	23.0
**Disability**	
No	57.0
Yes	36.0

Note: * mean; Some categories exceed 100 as participants were able to select all options that applied.

## Data Availability

The data that support the findings of this study are available on request from the co-authors, Mary Anne Adams and Tonia Poteat. The data is not publicly available due to it containing information that could compromise the privacy of research participants.
